# Psychological factors substantially contribute to biological aging: evidence from the aging rate in Chinese older adults

**DOI:** 10.18632/aging.204264

**Published:** 2022-09-27

**Authors:** Fedor Galkin, Kirill Kochetov, Diana Koldasbayeva, Manuel Faria, Helene H. Fung, Amber X. Chen, Alex Zhavoronkov

**Affiliations:** 1 Deep Longevity Limited, Hong Kong, People's Republic of China; 2 Department of Psychology, Stanford University, Stanford, CA 94305, USA; 3 Department of Psychology, The Chinese University of Hong Kong, Hong Kong, People's Republic of China; 4 Insilico Medicine, Hong Kong, People's Republic of China; 5 Buck Institute for Research on Aging, Novato, CA 94945, USA

**Keywords:** psychological aging, lifespan psychology, aging clocks, longevity

## Abstract

We have developed a deep learning aging clock using blood test data from the China Health and Retirement Longitudinal Study, which has a mean absolute error of 5.68 years. We used the aging clock to demonstrate the connection between the physical and psychological aspects of aging. The clock detects accelerated aging in people with heart, liver, and lung conditions. We demonstrate that psychological factors, such as feeling unhappy or being lonely, add up to 1.65 years to one’s biological age, and the aggregate effect exceeds the effects of biological sex, living area, marital status, and smoking status. We conclude that the psychological component should not be ignored in aging studies due to its significant impact on biological age.

## INTRODUCTION

Aging clocks are statistical models that enable measurements of biological age, as opposed to chronological age. While the latter is determined by one’s date of birth, the former depends on the intensity of aging processes and can be affected by genetics, life choices, and the environment. Most commonly, such aging clocks are regressors, trained to predict a person’s chronological age based on a vector of input parameters, such as clinical blood test results, gene expression levels, or DNA methylation intensities [1–5]. Recently, aging clocks linked to frailty and mortality by design have been trained [6, 7].

Based on the pace of aging, people may be divided into slow, normal, and fast agers. Several studies have indicated that increased biological age is associated with higher all-cause mortality, vulnerability to infection, and a number of non-communicable diseases [1, 2, 6, 8]. Conversely, a slow aging rate is associated with better health compared to age peers. Since aging clocks can detect various health conditions as accelerated aging, this technology holds the promise of becoming an all-purpose diagnostic and prognostic tool to benefit multiple healthcare-adjacent industries, such as insurance and nutrition. Aging clock studies are essential for the development of longevity therapeutics and geroprotectors (anti-aging medication). Aided by AI algorithms, aging clocks have the potential to become a staple of the modern drug design pipeline [5, 9–11]. This synthetic technology, which combines data-driven approaches, aging clocks, and deep learning, is being actively patented for use cases in global healthcare systems and the pharmaceutical industry [3, 12–17]. Longitudinal biogerontological studies have highlighted the ways to manipulate the pace of aging via exercise, diet, or dietary supplements [18–21]. Some compounds extensively studied in this context show great promise on a global scale [22].

The impact of psychology on biological age, however, is a painfully understudied subject. In the 20th century, several personality development theories have been formed, such as Freud’s psychosexual development and Erikson’s psychosocial development [23–25]. While Freudian psychology is more focused on early-life development, the stages defined by Erikson describe personality evolution from childhood until old age. In the last three decades, many theories of aging have been proposed (see [26] for a review). In particular, in 1990s, Laura Carstensen proposed a theory, called socioemotional selectivity theory, that describes how individuals shift their motivations across adulthood [27]. The theory specifies how psychological factors such as emotions can affect aging. However, while human psychology is known to go through certain changes throughout the life span, few studies have attempted to describe these changes in the terms of biogerontology (i.e., using aging clocks) or determine their effect on physical health [28, 29]. A limited number of studies pointed out that psychological factors, such as stress [30], loneliness [31], mental health [32, 33] and negative perceptions of aging [34], could have significant influences on aging clocks derived from physical parameters. Other socio-psychological factors that might accelerate aging clocks include negative life events [35], modern lifestyles [19, 31, 36], and low socio-economic status [37].

A study involving baboons linked social stress caused by competition to reversible aging acceleration [38]. Similar findings have been observed for humans: lifetime accumulated stress can be responsible for up to 3.6 years of extra biological age [39]. These findings suggest that the effectors of stress in organismal aging are glucocorticoid hormones, known to be involved in memory and cognition. Stress-induced epigenetic changes by glucocorticoid signaling colocalize with aging-associated DNA methylation sites, which may promote arteriosclerosis and other health conditions. Other effectors of stress-related aging include oxidative damage and telomere shortening, which may add up to ten years of extra biological age [40]. These stress-induced deviations from the normal aging pattern may persist for years, as shown in studies of childhood trauma and post-traumatic stress disorder [30, 41, 42]. Importantly, there is reason to believe that acting upon these psychosocial domains may confer a longevity benefit, as some studies have found that intense relaxation and emotional coping skills modulate biological age metrics [43, 44].

The link between physical aging and psychology is not unidirectional, as some studies have indicated. People with a high pace of aging are more likely to develop psychopathologies in later life [33], whereas epigenetic age at birth has been implicated in childhood temperament development [45]. In most studies, however, uncovering the causal links is an impossible task due to their cross-sectional design. The task is even further complicated by the inherent ambiguity of the term “psychological aging.” Psychological age may refer to the mental state most typical for people of a certain age group. At the same time, psychological age is something that corresponds to one’s subjective perception of the passing time, framed within their experiences and expectations. Interestingly, psychological age may be associated with opposite psychological trends, depending on the definition. For example, people with prominent neurotic behavior are more prevalent among chronologically younger people, yet neurotic people perceive themselves to be older than they really are [46].

In this article, we continue exploring psychology in the context of aging. We have developed a novel aging clock based on blood panels from the China Health and Retirement Longitudinal Study (CHARLS) dataset in order to define biological age in a cohort of Chinese adults. We represent biological age as a function of various psychological and social variables to measure their effects on the pace of aging. We also showed that AI-based psychological aging clocks are viable across cultures, building on prior work based on a U.S. dataset (i.e., an individualist culture) and now a Chinese dataset (i.e., a collectivist culture) [46]. Finally, we demonstrate the biological relevance of the aging clock by analyzing its error distribution in people with certain diseases.

CHARLS is a nationwide study of Chinese population older than 45 years that features information on respondents’ social and economic statuses, health history, biometrics, and blood panels [47]. Since its inception, CHARLS has been used to study the effects of nutrition, social bonds and economic status on healthy aging [48–51]. These insights have been used to measure the impact of social and economic policies on the Chinese national longevity potential [52–58].

Among East Asian countries, China has the lowest portion of successful agers: 15.7% [59]. A successful ager is a person over 65 years old with no major disabilities and with normal cognitive function and social engagement. For comparison, the portion of successful agers among older adults is 29.2% in Japan and 25.5% in Korea. Yet, due to the large population of China, the number of persons over 65 years old in China is higher than such number in the entire Europe [60]. Understanding aging in China can thus provide important insights on aging in the world.

**Figure 1:**
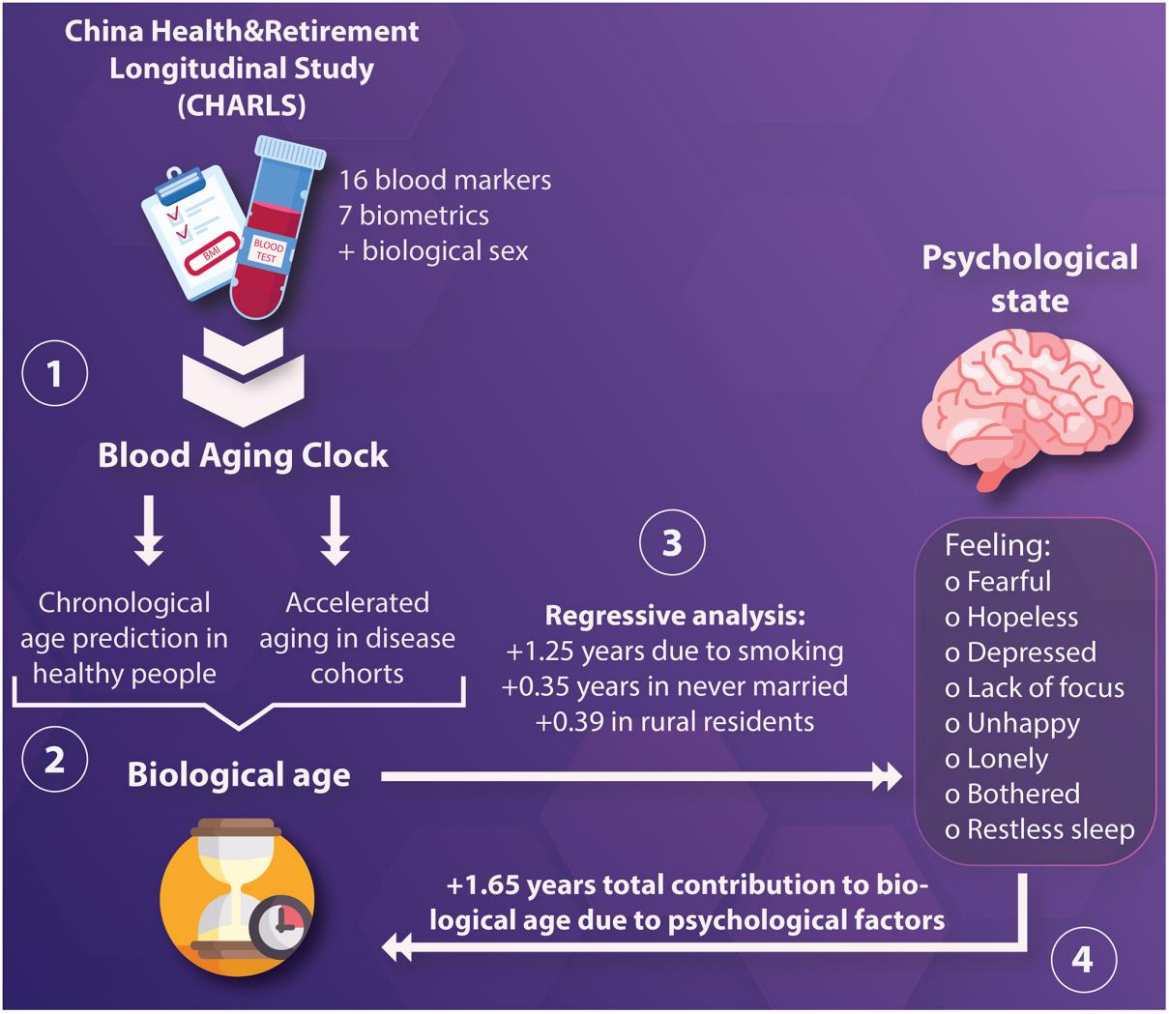
(**1**) Blood and biometric data, in addition to biological sex, were used to construct a neural network aimed to predict chronological age; (**2**) The age predicted by the model, hereby denoted as “biological age” was tested in healthy and ill participants to identify conditions interpreted as accelerated aging; (**3**) Then, regressive analysis was performed using an elastic net to quantify the total contribution of demographic, lifestyle, and psychological factors, hereby denoted as “psychological state,” to biological age; (**4**) The weight of each variable was understood as age acceleration, with the aggregate effect of one’s psychological state being able to accelerate biological aging by 1.65 years.

In this article, we demonstrate that mental and physical health are interlinked and both can be described by a single aging clock trained on blood test data (Figure 1). We also demonstrate that the detrimental impact of low psychological well-being is of the same magnitude as serious diseases and smoking.

## RESULTS

### Blood-based age predictor

**Figure 2:**
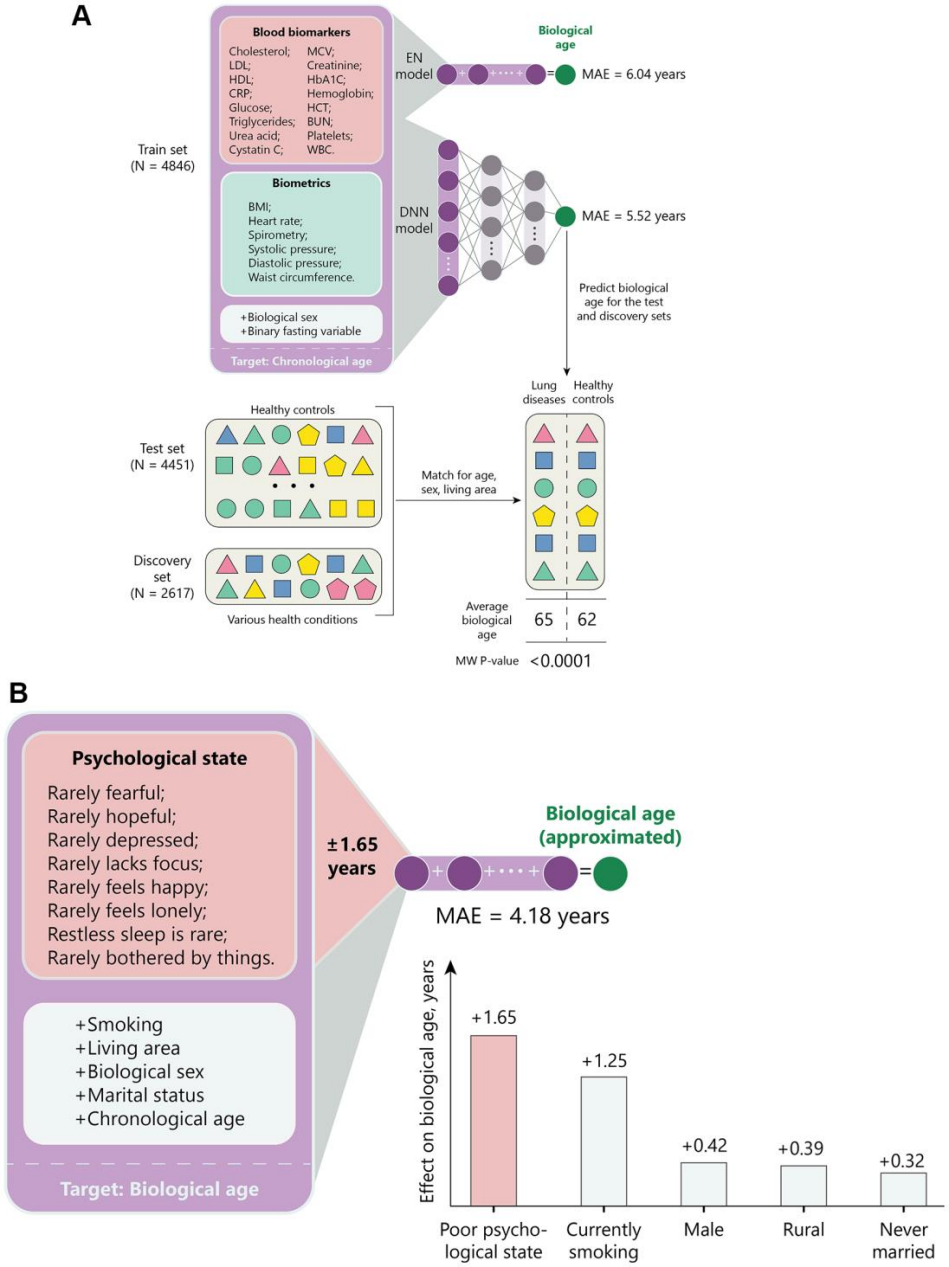
**Graphic description of the study.** (**A**) 4,846 entries from CHARLS were used to train a deep neural network regressor of chronological age, whose performance was compared to an elastic net model. The age predicted by a regressor is further referenced as “biological age”. Biological age of healthy (test set) participants and participants with a history of serious diseases (discovery set) was compared to identify conditions that are interpreted as accelerated aging by the model. To control for confounders, prediction averages were compared in matched cohorts. (**B**) The effect of psychological and other factors has been compared using elastic net. All variables, except chronological age, were converted to binary to predict biological age returned by the neural network described in panel (**A**). The weight of each variable was interpreted as age acceleration.

**Figure 3:**
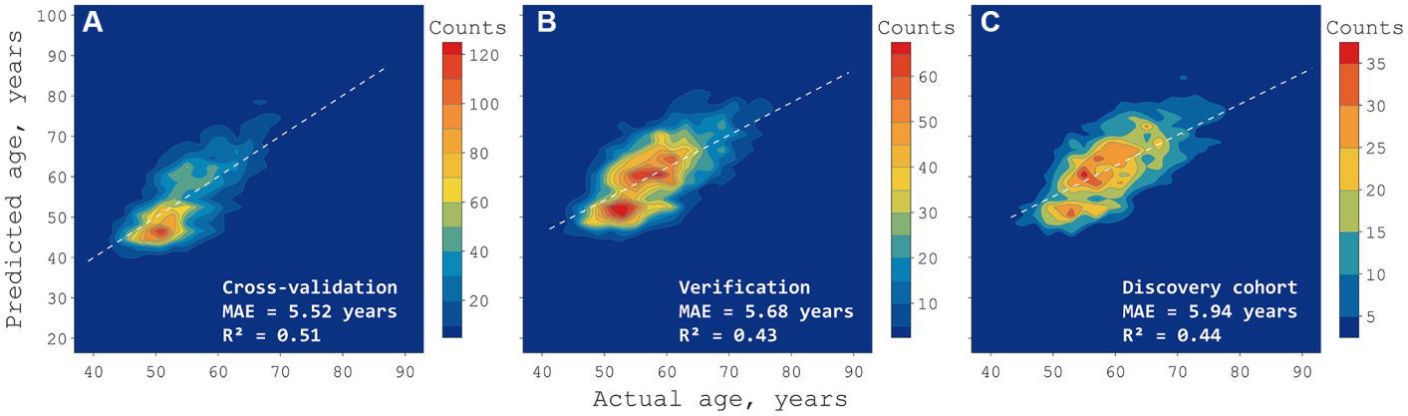
**Density map of deep neural network predictions in three sets.** The training set (**A**) was used to tune the neural network in a cross-validated manner, healthy samples from the test set (**B**) were used for verification, and the discovery set (**C**) contains only people with health conditions to explore cases of accelerated aging. The color bars mark continuous areas containing the same number of people. MAE stands for mean absolute error, R^2^ stands for coefficient of determination.

We used a combination of 16 blood biomarkers, seven biometric parameters, and the biological sex of CHARLS participants (see “Model Training”, Figure 2A) to create a deep neural network (DNN) age predictor. The model outperformed mean age assignment in both cross validation (CV) and test sets, as well as the discovery set comprised of participants with a history of serious health conditions (Figure 3, Table 1). To be more specific, we report a mean absolute error (MAE) of 5.68 years in a collection of 4451 test samples from generally healthy people.

**Table 1:** Deep neural network prediction of the chronological age of CHARLS participants both in cross-validation and test sets.

	**Metric**	**Cohort**		
		**CV**	**Test**	**Discovery**
**Mean age assignment**	**MAE, years**	8.38	7.11	7.32
**Elastic net**	**MAE, years**	6.04	6.19	8.06
**Neural network**	**MAE, years**	5.52	5.68	5.94
	**MAPE, years**	9.88	9.84	10
	**R^2^**	0.51	0.43	0.44
	***N*, people**	4846	4451	2617

The baseline model is mean age assignment. Abbreviations: CV: cross-validation; MAE: mean absolute error; MAPE: mean absolute percentage error; R^2^: coefficient of determination.

To illustrate the benefit of the deep learning approach, we also trained an elastic net (EN) using the same set division and variables and obtained a model displaying an MAE of 6.19 years for the same 4,451 people.

Based on the results shown in Supplementary Figure 1, the five most important features of our predictor are systolic and diastolic blood pressure, cystatin C, BMI and spirometry. We used permutation feature importance technique to measure feature weights for age prediction. The permutation feature importance (PFI) for a specific feature is defined to be the decrease in MAE when this specific feature column is randomly shuffled [61].

### Biological relevance of the predictor

To verify that the predictions of the new deep learning aging clock represented the pace of aging, we investigated whether people with aging-associated diseases were predicted to be older than healthy people.

The discovery set defined before training contained people with a history of cancer, heart attack, stroke, liver disease, or lung disease, while all participants in the test set had none of these conditions. To minimize the confounding effect of other variables, we analyzed isolated conditions (i.e., no comorbidities) juxtaposed against a subsample of healthy people with matching age, sex, and living area distributions.

People with a history of stroke, liver disease, and lung conditions were predicted to be significantly older (*P* value < 0.0005, *U* test) than their healthy counterparts (Table 2). However, the mean effect of these conditions on the predicted age did not exceed +1.50 years, which is below the model’s MAE of 5.56 years.

**Table 2:** Differences in deep neural network age predictions between afflicted people in the discovery cohort and healthy people in the test cohort.

**Disease**	**Afflicted error, years**	**Healthy error, years**	**Delta, years**	***N*, people**	**Median age, years**	***P* value**
Heart disease	0.427	0.459	−0.03	873	63	0.873
Cancer	−0.333	0.74	−1.07	73	59	0.225
Stroke	1.557	0.068	1.49	115	65	^*^0.0156
Lung disease	1.869	−0.336	2.2	782	63	^*^<0.0001
Liver disease	0.138	−0.829	0.97	280	59	^*^0.0166

The comparison has been carried out in samples matched for age, sex, and living area (rural or urban). “^*^” marks the diseases with significantly accelerated pace of aging (*P* value < 0.05, Mann-Whitney *U* test).

No significant differences in age predictions were detected for people with a history of heart disease or cancer when compared to healthy controls. It should be noted, however, that cancer is the least frequent condition in CHARLS and the lack of significance may also have been caused by insufficient sample sizes.

### Effects of psychological states on the pace of aging

To compare the effect of multiple factors on biological age, we used EN to regress aging clock’s predictions as a function of demographic and psychological variables (Figure 2B). All factors included in the analysis (except for being widowed) showed significant effects on the pace of aging (Table 3). The three largest effects on the aging rate belonged to the following binary factors: smoking (+1.25 years), being currently married (−0.59 years), and suffering from restless sleep (+0.44 years). However, if the eight psychological variables are considered as a scale representing psychological well-being, being on its lower end has an effect of accelerating aging by 1.65 years.

**Table 3:** Biological age obtained from the deep neural network predictor was regressed as a function of chronological age and additional features to estimate their effect on the pace of aging.

**Type**	**Variable**	**Coefficient**	**Standard deviation (σ)**
**Demographic**	**Chronological age, years**	0.49	0.00
	**Is male**	0.42	0.02
**Social**	**Is married**	−0.59	0.04
	**Is widowed**	0.27	0.09
	**Is rural**	0.39	0.01
**Psychological**	**Rarely bothered by things**	0.09	0.03
	**Rarely lacks focus**	−0.05	0.02
	**Rarely depressed**	−0.09	0.02
	**Rarely hopeful**	0.28	0.01
	**Rarely fearful**	−0.29	0.02
	**Restless sleep is rare**	−0.44	0.01
	**Rarely feels happy**	0.35	0.02
	**Rarely feels lonely**	0.05	0.03
**Smoking**	**Currently smoking**	1.25	0.02
	**Intercept**	28.37	0.02
**Metrics**			
	**MAPE (CV), %**	7.57	
	**MAPE (Test), %**	6.37	
	**MAE (CV), years**	4.18	
	**MAE (Test), years**	4.09	
	**N (CV), people**	4846	
	**N (Test), people**	4451	

All variables, except for chronological age, are binary. All coefficients, except for “Is widowed”, are significant within 3σ, as estimated with ten random cross-validation splits. Abbreviations: CV: cross-validation; MAE: mean absolute error; MAPE: mean absolute percentage error.

## DISCUSSION

In this article, we have demonstrated a novel blood aging clock developed using only Chinese data from CHARLS. Previous installments of BloodAge have been validated in Russian, Canadian, European, and Korean populations [1, 62]. The same technology has also been utilized in numerous settings, including mortality-linked datasets [1, 8, 63]. In these studies, BloodAge has demonstrated strong population-specific effects, with some nations or races showing significantly different error distributions. Thus, to ensure that biological age can be accurately estimated in the Chinese population, we trained a new DNN with CHARLS data.

While CHARLS contains an abundance of medical and demographic profiles, blood panels are available in two of its waves. We chose the 2015 wave due to a larger number of measured blood parameters, yet it is still below the number of biomarkers available in other public datasets, such as NHANES. While the original BloodAge was trained on blood panels with 46 biomarkers from over 60,000 people, CHARLS offers only 16 blood parameters [62]. Our initial experiments with this limited variable space failed to produce an accurate predictor of chronological age; hence, we augmented the training data with biometric data, such as BMI, waist circumference, and blood pressure. In total, we were able to train a DNN with 24 variables and 4,846 healthy samples to reach an accuracy of MAE = 5.68 years, which is almost identical to 5.55 years in the original BloodAge publication from 2016 [62].

We proceeded to validate the output of the DNN as a general health marker for a subset of CHARLS subjects with known health issues (Table 2). Our hypothesis was that people with aging-related diseases should have DNN prediction errors shifted to the right, thus indicating the increased pace of aging. To correct for confounders, we compared the errors within subsets of people afflicted by only one condition and healthy subsets matched for sex, age, and living area. Among the five conditions, significant age acceleration was detected for the participants with a history of stroke, liver, and lung conditions. At the same time, people who had suffered from heart attack or cancer had a DNN error distribution indistinguishable from healthy controls. It should be noted that the lack of significance for cancer may have been caused by the heterogeneity of oncological diagnoses, as well as seemingly anti-aging properties of malicious cell lines (such as proliferation) [64].

The health conditions accelerated the aging processes by 1–2 years. Given the high average age of the afflicted participants, this seemingly minor age acceleration can lead to a significant increase in mortality risk. The effect of age acceleration on survival, however, cannot be assessed in the absence of mortality-linked data. Altogether, we consider that the described aging clock possesses the property of disease-relevance that makes its prediction error representative of a participant’s health.

Since accelerated aging was demonstrated in cohorts matched for age, sex, and living area, this effect offers additional predictive value compared to chronological age. However, an increased pace of aging may have multiple causes and thus lacks specificity. This drawback may be eradicated in future ensemble models that classify inputs in disease-specific categories and return biological age as a metric for severity. Among the blood biomarkers included in the DNN aging clock, cystatin C was the second most important, ranking below systolic pressure. Cystatin C is a cysteine protease used as a biomarker of renal function and its elevation is a major risk factor in all-cause mortality in the Chinese older adults [65]. Some other studies of mental and physical health have linked abnormal cystatin C levels to new-onset depressive symptoms [66].

Finally, we quantified the effect of psychological features on the pace of aging with the EN approach. The biological age estimate, obtained with the DNN, was regressed as a function of chronological age, sex, marital status, living area, and several mental state variables (Table 3). We deliberately chose not to include biometric features used by the DNN since they are implicitly present in the biological age target itself.

To make the interpretation easier, we converted all variables, except for chronological age, into a binary form. Since CHARLS contains mostly middle-aged or older participants, the EN features a high intercept (28.37 years), and its results may not be applicable to younger datasets. According to the EN interpretation, the biological age of any person is almost fully comprised of the intercept and chronological age. Nonetheless, most other features had significant non-zero coefficients, as estimated with ten-fold CV. In aggregate, the psychological state of a participant contributes 1.65 years of biological age if all the associated variables assume the values indicating lower well-being. For example, feeling unhappy will contribute 0.35 years and having no trouble with sleep will detract 0.44 years.

The survey collection method may have affected the significance of the psychological factors. The participants were asked to rank the frequency of certain feelings or issues during the past week. We believe that in more thorough assessments of psychological well-being, the contribution of such features would be larger.

The impact of demographic features is of a similar magnitude as that of psychological factors. Among them, marital status had the greatest effect on the pace of aging. Currently married participants were predicted to be 0.6 years younger than the never married, while widowed participants were predicted to be 0.3 years older. Previous CHARLS-based studies showed that widowhood was associated with signs of accelerated aging, particularly memory decline [50, 51]. However, the data did not allow us to study marital satisfaction as an aging factor, although we hypothesize that it positively correlates with the other quantified psychological effects and thus is beneficial for the pace of aging [67, 68]. We also interpret this finding as the importance of family support in older age. Both instrumental and emotional support from the spouse and offspring significantly improve life satisfaction, and thus decrease the aging rate [69]. At the same time, feeling lonely had a nonsignificant effect on the pace of aging, indicating that family support is much more important for successful aging than weak ties with low emotional intensity [49]. However, some studies have reported that the Chinese older adults (except the oldest old) display slightly lower life satisfaction scores if they still live with their children [70]. Altogether, these findings show that family support may improve longevity potential only in combination with a reasonable amount of autonomy, although there might be cross-cultural differences.

Additionally, people living in rural areas are predicted to be 0.4 years older than those living in urban areas. The adverse effect of rural living has been recently shown in a study of multimorbidity in China and has been attributed to “lower socio-economic status, poorer social services, and lower access to quality medical services” [71]. Malnutrition and physical labor are proposed as the key contributors to the increased prevalence of certain conditions, such as osteoporosis and arthritis. These factors are also contributing to poorer mental health in the older Chinese rural population, who are four to six times more likely to be depressed that the urban population [72, 73].

The new DNN successfully represents both mental and physical health decline as cases of accelerated aging. This property makes this new aging clocks a great tool for developing anti-aging therapeutics. The impact of the interventions targeting the body may be assessed with the original DNN, while the efficiency of the interventions targeting one’s mental state may be estimated with the EN summarized in Table 3. Both modes of this new aging clock may be combined to construct personalized regimens reducing the pace of aging. The utility of this aging clock may be further increased by combining it with a SOM-based recommendation engine we presented earlier this year, FuturSelf [74]. This model of human psychology is available at https://futurself.ai/ and provides personalized recommendations aimed to maximize users’ future mental well-being.

We are planning to validate this approach in other public datasets before releasing the model. However, the exact wordings for the psychological assessment in CHARLS make the model less compatible with Western datasets, such as UK Biobank or NHANES.

Altogether, we have demonstrated that the pace of aging is significantly associated with psychological features. The observed contribution of one’s mental state to one’s biological age is significant and is comparable to the effect of smoking. Thus, promoting mental health may be considered a potential anti-aging intervention with possible benefits at par with more tangible, physical therapeutic approaches.

## CONCLUSION

Using data from the Chinese CHARLS database, we have demonstrated that organismal aging is not only determined by physical factors but also, to a certain degree, affected by mental state and social status. We interpreted biological age as a proxy for the general state of health and show that positive feelings (happiness, hope, safety) have a significant impact on the former.

The study findings further support the necessity of companionship and a psychologically pleasant environment for healthy longevity.

## METHODS

### Data overview

This study features data from CHARLS collected in waves between 2008 and 2018, with two waves (2011, 2015) featuring blood panels. We used data from the 2015 wave due to a larger number of available blood biomarkers. In total, 11,914 entries were imported from CHARLS and divided into training (*N* = 4,846), test (*N* = 4,451), and discovery (*N* = 2,617) sets. Samples with missing sex or outlier values in the predictor variables were excluded. The training set comprised generally healthy people. The test set aggregated healthy people and people with the following health issues: hypertension (*N* = 1,277), arthritis (*N* = 915), dyslipidemia (*N* = 278), kidney disease (*N* = 207), and diabetes (*N* = 115). The discovery set contained only samples with a history of cancer (*N* = 73), chronic heart condition or heart attack (*N* = 873), stroke (*N* = 115), lung disease (*N* = 782) or liver conditions (*N* = 280).

The distributions of all CHARLS variables mentioned in this article are available in Supplementary Tables 1, 2 and Supplementary Figure 2.

### Model training

To train the predictor, we used 16 blood panel variables: total cholesterol, LDL cholesterol, HDL cholesterol, C-reactive protein, glucose, triglycerides, urea acid, cystatin C, MCV, creatinine, HbA1C, hemoglobin, hematocrit, BUN, platelet count, and WBC count. These variables were further complemented with biological sex, a binary variable indicating whether the person was fasting before blood drawing, and six biometric variables: systolic and diastolic pressure, BMI, waist circumference, heart rate, spirometry. In cases of CHARLS participants having multiple measurements for a biometric parameter, the average was used.

We used feed-forward DNN with more than three hidden layers for the age prediction task. We treated age prediction as a regression task, so linear activation function was used on the DNN output. All features were normalized and mapped to the 0–1 space using MinMaxScaler from the scikit-learn Python package in order to prevent gradient explosion and make all features equally important for the DNN [75, 76]. The MAE loss function between real and predicted age was used as an objective and minimized during training. Grid search was applied to find the best model parameters in terms of an objective metric (MAE). The best performing model had the following parameters: 5 hidden layers with 256 neurons each, an exponential linear unit (ELU) activation function after each layer, dropout with 35% probability, and L2 with coefficient 1e-5 after each layer for the purpose of regularization [77, 78]. All models were trained until convergence on the validation set (when loss on validation set stopped decreasing).

The DNN was trained using five-fold CV to compensate for overfitting and obtain more robust performance metrics. We used Python 3.9 along with the Keras library (https://keras.io/) with a Tensorflow backend (https://www.tensorflow.org), to build and train the DNN.

The metrics used to measure the performance of the models were MAE, mean absolute percentage error (MAPE), and coefficient of determination (R^2^). These metrics are defined as follows:


$$\begin{array}{l} MAE=\frac{1}{N}\overset{N}{\underset{i=1}{\sum }}\left|Age_{true,i}-Age_{predicted,i}\right|,\\ \;\;\;\;\;\;\;\;\;\;\;\;\text{where}\;N\;\text{is}\;\text{the}\;\text{total}\;\text{number}\;\text{of}\;\text{samples} \end{array}$$

$$\begin{array}{c} MAPE=\frac{100}{N}\overset{N}{\underset{i=1}{\sum }}\frac{1}{Age_{true,i}}\left|Age_{true,i}-Age_{predicted,i}\right|,\\ \;\;\text{where}\;N\;\text{is}\;\text{the}\;\text{total}\;\text{number}\;\text{of}\;\text{samples} \end{array}$$

$$R^{2}=1-\frac{\sum_{i}Prediction\;error_{i}^{2}}{N\times D\left(Age_{true}\right)},\;\text{where }D\text{ is the variance}$$

The performance of our DNN model was compared against mean age assignment and an EN model trained using the ElasticNet method from the sklearn. linear_model module for Python. The EN was trained on the same training set and features as the DNN. The EN’s hyperparameters were optimized using the following grid: {‘alpha’: [1e-5, 1e-4, 1e-3, 1e-2, 1e-1, 0.0, 1.0, 10.0, 100.0], ‘l1_ratio’: np.arange (0, 1, 0.01)}.

### Association with health conditions

Age prediction errors were compared between people with a history of cancer, stroke, heart attack, liver disease, or lung disease from the discovery set and healthy participants from the test set. To correct for biases associated with cohort differences, we matched disease-associated subsamples with equally sized subsamples of the test set for age, sex, and living area.

The three meta variables were normalized to generate matched healthy cohorts with the nearest neighbor approach. The test set was sampled with replacement for each condition. The Mann-Whitney *U*-test was carried out for each condition to establish the significance of different aging rates.

The nearest neighbor search was implemented with NearestNeighbors from scikit-learn 0.24.2, and the *U*-test was implemented with mannwhitneyu from scipy 1.7.0 for Python 3.9.

### Association with psychosocial factors

To establish the effect of multiple variables on the pace of aging, we used EN regression. The predictions of the deep learning aging clock were regressed as a linear combination of chronological age, sex, marital status, living area, smoking status, and eight psychological attributes (Table 3). All variables, except for chronological age, were binarized.

The coefficients of the final EN regression represent the effect of each associated parameter on the pace of aging. Standard deviations of the coefficients were calculated based on ten iterations with different random seeds. The EN model was trained using the 6,511 training samples and verified with 2,786 samples from the test set.

## Supplementary Materials

Supplementary Figures

Supplementary Tables
