# Supplementary material for: Psychological factors substantially contribute to biological aging: evidence from the aging rate in Chinese older adults
Source: Aging (Albany NY). 2022 Sep 27;14(18):7206–22. doi: 10.18632/aging.204264 (PMC9550255; doi:10.18632/aging.204264)
Supplement: Supplementary Figures [file aging-14-204264-s001.pdf]

SUPPLEMENTARY FIGURES

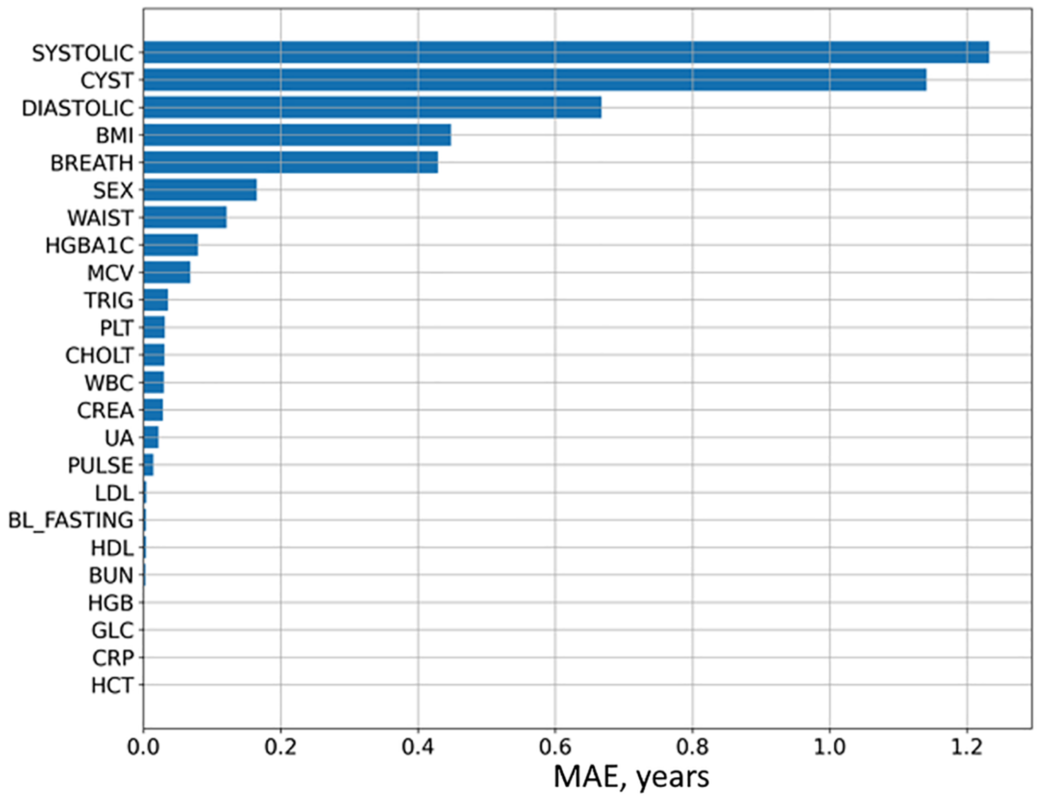

Supplementary Figure 1. Permutation feature importance analysis was carried out to determine the features our aging clock pays the most attention to during age prediction. The importance of a feature is judged based on the drop in the MAE of the model when a feature's value is shuffled in the test set.

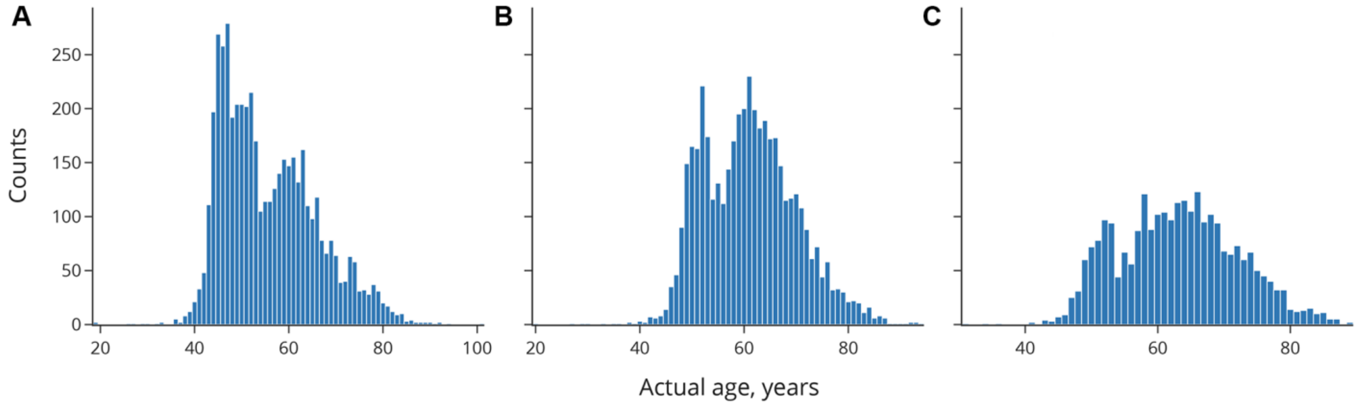

Supplementary Figure 2. Age distribution in the three cohorts used in this study. (**A**) Training set (*N* = 4846), mean age is 55.7 years (**B**) Test set (*N* = 4451), mean age is 61.0 years (**C**) Discovery set (*N* = 2617), mean age is 63.0 years.
