# Supplementary material for: Psychological factors substantially contribute to biological aging: evidence from the aging rate in Chinese older adults
Source: Aging (Albany NY). 2022 Sep 27;14(18):7206–22. doi: 10.18632/aging.204264 (PMC9550255; doi:10.18632/aging.204264)
Supplement: Supplementary Tables [file aging-14-204264-s002.pdf]

SUPPLEMENTARY TABLES

Supplementary Table 1. The prevalence of the conditions used in the elastic net importance analysis section.

Feature	Percent positive
**Is male**	45.90%
**Is married**	89.15%
**Is widowed**	9.25%
**Is rural**	73.93%
**Current smoker**	28.37%
**Bothered by things is rare**	55.45%
**Lack of focus is rare**	56.53%
**Depression is rare**	55.43%
**Hopeful is rare**	29.32%
**Fearful is rare**	83.32%
**Restless sleep is rare**	53.14%
**Happiness is rare**	19.05%
**Loneliness is rare**	74.88%
**Total *N***	9297

The statistics are derived from the training and test sets only.

Supplementary Table 2. The mean values and standard deviations (std) of the variables used by the age predictor.

Variable name	Mean	Std	Units
**White blood cells**	5.98	2.06	x10E9/L
**Hemoglobin**	137.28	19.22	g/L
**Hematocrit**	41.50	5.58	%
**Mean corpuscular volume**	91.28	7.70	fL
**Platelets**	205.31	74.14	x10E9/L
**Triglycerides**	1.61	1.03	mmol/L
**Creatinine**	70.96	24.05	umol/L
**Blood urea nitrogen**	5.48	1.63	mmol/L
**HDL cholesterol**	1.32	0.29	mmol/L
**LDL cholesterol**	2.64	0.74	mmol/L
**Total cholesterol**	4.75	0.93	mmol/L
**Glucose**	5.70	1.87	mmol/L
**Uric acid**	0.29	0.08	mmol/L
**Cystatin C**	0.84	0.23	mg/L
**C reactive protein**	2.57	5.31	mg/L
**Glycated hemoglobin**	5.96	0.96	%
**Systolic pressure**	127.59	19.64	mmHg
**Diastolic pressure**	75.61	11.63	mmHg
**Heart rate**	73.98	10.53	bpm

**BMI**	23.95	3.63	kg/m2
**Spirometry**	296.03	117.63	L/min
**Waist circumference**	86.36	10.4	cm
**Total *N***		11914	

The statistics are calculated based on the total study sample including training, test, and discovery sets.
